# Mapping mental health finances in Ghana, Uganda, Sri Lanka, India and Lao PDR

**DOI:** 10.1186/1752-4458-4-11

**Published:** 2010-05-27

**Authors:** Shoba Raja, Sarah K Wood, Victoria de Menil, Saju C Mannarath

**Affiliations:** 1 Policy and Practice Directorate, BasicNeeds, 114, 4th Cross, OMBR Layout, Banaswadi, Bangalore, India; 2 BasicNeeds, 158 A Parade, Leamington Spa, Warwickshire, UK

## Abstract

**Background:**

Limited evidence about mental health finances in low and middle-income countries is a key challenge to mental health care policy initiatives. This study aimed to map mental health finances in Ghana, Uganda, India (Kerala state), Sri Lanka and Lao PDR focusing on how much money is available for mental health, how it is spent, and how this impacts mental health services.

**Methods:**

A researcher in each region reviewed public mental health-related budgets and interviewed key informants on government mental health financing. A total of 43 key informant interviews were conducted. Quantitative data was analyzed in an excel matrix using descriptive statistics. Key informant interviews were coded *a priori *against research questions.

**Results:**

National ring-fenced budgets for mental health as a percentage of national health spending for 2007-08 is 1.7% in Sri Lanka, 3.7% in Ghana, 2.0% in Kerala (India) and 6.6% in Uganda. Budgets were not available in Lao PDR. The majority of ring-fenced budgets (76% to 100%) is spent on psychiatric hospitals. Mental health spending could not be tracked beyond the psychiatric hospital level due to limited information at the health centre and community levels.

**Conclusions:**

Mental health budget information should be tracked and made publically accessible. Governments can adapt WHO AIMS indicators for reviewing national mental health finances. Funding allocations work more effectively through decentralization. Mental health financing should reflect new ideas emerging from community based practice in LMICs.

## Background

Although not the only obstacle, one of the primary barriers to adequate mental health care is inappropriate mental health financing [1,2]. In ten years of delivering mental health and development services across eight low and middle income countries, BasicNeeds and its 42 implementing partners, from both government and community sectors, have been challenged by the scarcity of resources. Our government partners, particularly those within health ministries, share in the desire for greater capacity - both human and material - so they can deliver the mental health services being demanded by thousands of individuals and their families.

The World Health Organisation (WHO) asserts that "without adequate financing, mental health policies and plans remain in the realm of rhetoric and good intentions" [3]. This bold stance sets the tone for the report *Mental Health Financing*, which constitutes one module of the *Mental Health Policy and Service Guidance Package *issued by the WHO in 2003. The guidance package lays out eight sequential steps to good mental health financing. The first two steps are to understand the broad health financing context, and to map current resources and how they are used in the mental health system.

### WHO ATLAS Data (Global & Regional Levels)

Following its own guidance, the WHO preliminarily mapped national budgets for mental health as one component of its ATLAS project, which documents all mental health resources globally [4]. ATLAS questionnaires were sent to a mental health focal point at the Ministry of Health in 191 countries. The questionnaires contained three queries relating to mental health financing: 1) Is there a national budget line for mental health? (and if so, how much?); 2) How are mental health services financed?; and 3) Is mental illness considered a disability for public disability benefits?

The results of ATLAS study, particularly in answer to the first question about budgeting, gave the first good picture of the status of mental health financing globally. Out of all the countries surveyed, one in three (32%, n = 61) had no specific budget for mental health [5]. Although 130 countries reported having a mental health budget, only 89 of them were able to provide information about their mental health budget. Of those 89 providing budgetary information, one in three (36%, n = 32) spent less than 1% of their health budget on mental health.

**Figure 1:**
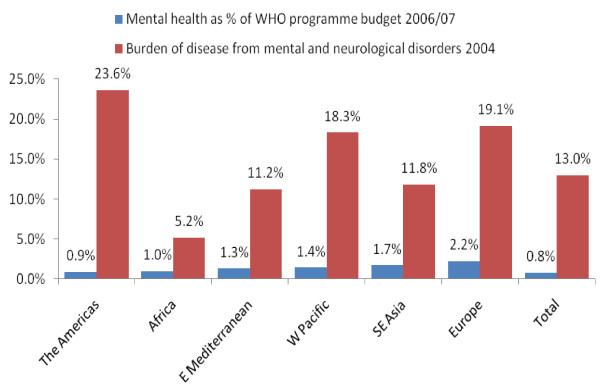
**Neuropsychiatric disorders as a percent of WHO programme budget vs. percent of disease burden**.

The ATLAS findings on the small government allocations for mental health are corroborated by data on the WHO's mental health budget. In 2006/07 the WHO allocated only 0.8% of its total operating budget to mental health, amounting to a total of US $14.9 million per year [6], despite that neuropsychiatric disorders represents 13% of disease burden [7]. Since the WHO reflects the priorities of its member states, their budget serves as further evidence that mental health financing is not yet a priority for most countries (refer to figure 1).

Regionally, those spending proportionally the least on mental health in the ATLAS study were predominantly located in Africa and South-East Asia. Seventy nine percent (79%) of African countries and 63% of Asian countries spent less than 1% of their health budgets on mental health [6]. Given that these regions encompass some of the lowest income countries in the world, 1% percent amounts to a small portion of a small pie.

That 36% of countries spend less than 1% of their health budgets on mental health, when mental disorders represent 13% of the global burden of disease, points to a striking disconnect between disease burden and health spending. More striking is that 53% of countries (n = 102) provided no information about spending on mental health [6]. This finding suggests one of three possibilities: 1. no information was available about mental health financing in these countries; 2. Information about mental health financing exists but was not transparent; 3. mental health was not a high enough priority for ministries to respond to the WHO survey. In either case, it is clear that more research is needed into mental health financing in low income countries.

### WHO AIMS Data (Country Level)

Building on the preliminary evidence from the ATLAS study, in 2005 the WHO launched a new tool, called the Assessment Instrument for Mental Health Systems (AIMS) [8]. Unlike ATLAS, which was collected from all countries for the purpose of creating a global evidence base about mental health resources, AIMS is collected only by interested countries at the request of their health ministry with technical support from the WHO. Considerably more thorough than ATLAS, the AIMS process involves contacting people from many levels within the health system and takes an estimated 6 months to conduct, including quality checks from the WHO regional office. The AIMS tool is constructed along six domains, and mental health financing constitutes one facet of the domain on policy and legislation. Where ATLAS collected information on three financing indicators, six indicators are collected by AIMS, namely: 1) mental health expenditures by the government health department; 2) expenditures on mental hospitals; 3) mental disorders in social insurance schemes; 4) free access to essential psychotropic medicines; 5) affordability of antipsychotic medication; and 6) affordability of antidepressant medication.

In a first-of-its-kind study, Daniel Chisholm and colleagues recently estimated the cost of implementing a core package of mental health services at a high level of coverage in low and middle income countries [9]. Drawing data from the AIMS profiles of 12 countries, they calculated the cost of achieving 80% coverage for schizophrenia and bipolar disorder and approximately 30% coverage for depression and problem drinking. They based their estimates on evidence-based interventions offered predominantly in primary care. The conclusion of these health economists was that it would cost low-income countries a minimum of $1.85 per capita to provide this core package of services at the desired volume.

### Limited Mental Health Financing Information

Outside of the previously mentioned WHO studies, limited evidence exists about mental health financing in low and middle-income countries. A lack of transparency about mental health funding sources and allocations in LMICs contributes to this knowledge gap. According to Knapp et al., mental health financing challenges may include: insufficiency, poor distribution, inappropriateness, inflexibility, poor coordination and timing of disbursement [10]. However, it is important to identify whether these barriers are applicable to individual countries or regions.

A 2007 policy brief titled *Developing Effective Mental Health Policies and Plans in Africa *analyzed mental health care policies in Africa (Mental Health and Poverty Project, unpublished). They found many of these policies lacked any clear information about how broad objectives can be achieved within available resources. While mental health policies for South Africa, Ghana [11] and Zambia refer to financing, no specific mention is made for the source or allocation of funds. The Uganda mental health policy does not mention financing at all. Likewise, a 2007 study titled *Mental health: access to treatment and macroeconomics in Ghana *found that unclear national mental health priorities contributed to inadequate funding for mental health services (Appiah Kubi et al., unpublished). This pressing need for mental health financing information in Africa has clear policy implications for individual countries.

Access to psychiatric medicines in Africa is hampered by inadequate supply and the cost of out-of-pocket payments for medicines [12]. Some countries, such as Ghana, rely completely on donor funding for psychiatric medicines. When medicine is unavailable in the public sector, prescribers, dispensers and users turn to the private sector for needed psychiatric drugs. A 2006 comparative analysis on the affordability of chronic disease medication revealed that the lowest paid unskilled government worker in Kenya would need to work 20.2 days to pay for one month's worth of generic fluoxetine [13]. However, government financing of psychiatric medicines has not yet been explored in depth.

The three new WHO resources - the guidance package, ATLAS and AIMS - have grounded the field of global mental health with a baseline of financing data and a clear process for strengthening financing systems. Nonetheless, these tools have certain limitations, as recognised by the WHO themselves. The ATLAS data provides a good picture at global level, however at country level, its financing data is of questionable accuracy. In the words of its lead investigators, "Some countries were not able to give information on certain themes, often because such data simply do not exist within the countries." In addition, ATLAS only collected data at the national level, whereas many countries have devolved the bulk of their health budgets to states, provinces or regions.

AIMS is a stronger tool for national mental health financing, however, it is not available for all countries. At the time of writing, two thirds of WHO member states have not yet completed the AIMS study. In addition, both AIMS and ATLAS data is only presented proportionally, in terms of percentages; no absolute numbers on health spending are available through the WHO. While it is useful for the purpose of comparison to know that 1% of a health budget is spent on mental health, it is important to know what that 1% amounts to when trying to resource a health system within country.

It is in this context of limited information about public funding of global mental health that the international NGO BasicNeeds undertook a study into the financing of mental health in five of the eight countries where it operates. BasicNeeds began operations in 2000 with the purpose of enabling people with mental illness or epilepsy to live and work successfully in their communities. From its establishment through December 2009, BasicNeeds had worked with a cumulative total of 78,036 people with mental or neurological disorders, of whom 33,915 are currently active in the programme [14]. The organisation implements a holistic intervention called the Model for Mental Health and Development, combining medical, social and economic activities as well as research [15]. The current study reports on qualitative and quantitative research into mental health financing in Ghana, Uganda, Sri Lanka, India (Kerala), and Lao PDR with an aim to better understand how much money is available for mental health in these countries, how it is spent, and how this impacts mental health services.

## Methods

This study utilizes document reviews and key informant interviews to explore mental health financing information in the public sector, including non-profit aid to the public sector. The study seeks to understand the following questions: 1) what funding is available for public mental health services; 2) how is the funding spent; and 3) how does mental health financing impact mental health services.

### Study Setting

The study was conducted in five countries - three low-income (Ghana, Uganda and Lao PDR) and two lower-middle income (India and Sri Lanka) (World Bank 2009) -- between May and October 2009. These study areas were chosen based on BasicNeeds' presence and contacts within government mental health services in the countries. All data were collected by five BasicNeeds Research Officers, who are nationals of the country in which they work.

### Key Informants

Data were collected through key informant interviews and document reviews. A purposively selected sample of key informants included both government administrators and clinicians at primary health care and referral levels. Key informants were identified from two levels: national and local. In some countries, the first round of key informants introduced the researcher to other informants. A total of 43 informants were interviewed for the study with a range of 6-13 per country (Sri Lanka 6, Uganda 6, India 7, Ghana 11, Lao PDR 13).

Consent was received before data collection in all five countries. In Sri Lanka, consent was written, whereas in the other countries oral consent was obtained. All interviews were confidential. None of the informants declined to be interviewed.

Two pre-designed interview guides were used to conduct the key informant interviews: one for government officials at the national level and the other for informants from regional referral hospital, districts and primary health care levels. In Kerala and Lao PDR, the interviews were conducted in the local language and translated by the researcher at the time of data consolidation. In Uganda, Sri Lanka and Ghana, interviews were conducted in English. The interviews were documented using audio devices in Sri Lanka and Lao PDR and written notes in other regions.

### Document Reviews

Documents were chosen based upon their relevance to health and mental health financing in each area. Key informants played an instrumental role in leading researchers to appropriate budget documents. The documents reviewed include annual reports of mental hospitals, primary health centres, and the health ministry. A Document Review Matrix was used to facilitate data collection about the size of mental health budgets and their allocation.

### Data Analysis

Data analysis was done at two levels: at the country level and internationally. Country researchers collected and analysed country data using *a priori *coding techniques to analyze the qualitative responses of key informants. The responses were coded according to predetermined research questions. The budget data was consolidated and analyzed using Excel. At the international level, two researchers consolidated the data from each country and performed additional calculations in Excel for cross-country comparison.

## Results

Findings are organized by country with the exception of India, for which data was only collected for the state of Kerala. Collecting national data for India was outside the scope of this study, and the state population size makes Kerala more suitable to comparison with the other countries.

### Ghana

#### Public Funding for Mental Health

**Figure 2:**
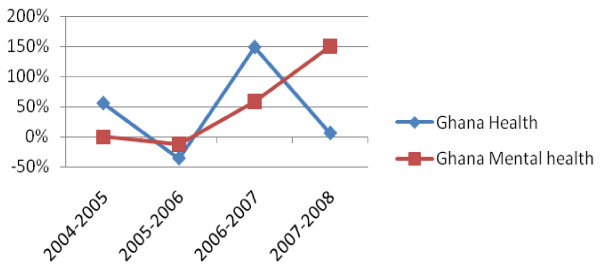
**Ghana % Change in Ring-Fenced Budget for Mental Health vs All Health 2004-2008**.

In Ghana, the ring-fenced budget for mental health care in 2007-2008 was $17,412,263, which breaks down to a per capita allocation of $0.76. These findings indicate that 3.9% of Ghana's health budget was ring-fenced to mental health in 2007-2008. As shown in Figure 2, Ghana's ring-fenced budget for mental health has seen a steady increase over the past five years, despite the erratic trends of the overall health budget.

#### Other Funding for Mental Health

In addition to funding from government, mental health in Ghana is also funded by general hospitals, through their internally generated funds, and by international donors. Internally generated funds are funds from fee-paying patients. The money is pooled into a communal hospital fund and distributed across services within the hospital. Mental health services do not usually generate revenue, since most service users are too poor to pay the fees. As a result, mental health services are subsidised by this system of pooling internally generated funds. As one hospital medical director explained:

"*As a hospital we run all services, including mental health. If we want to be programme oriented, then mental health will suffer because they do not generate any money but they are supported through our IGF [internally generated funds] from other health sectors. So the communal pool and consumption are a good advantage to mental health in that, here, the strong supports the weak."*

The amount of spending allocated to mental health through pooling of hospital funds was not possible to determine. Much of the money covers overhead costs, including basic medical supplies and facility maintenance.

The largest NGO involved in mental health in Ghana is BasicNeeds, which works across the three northern regions and Accra with a population of 16,024 people with mental illness or epilepsy and an estimated 8,000 carers [11]. The BasicNeeds Ghana budget in 2007-2008 was $651,660, which was funded predominantly by the UK Department for International Development, the European Commission Development Fund and Comic Relief, a private British foundation.

#### Budget Allocation

100% of the government's ring-fenced mental health budget in Ghana was allocated to 3 psychiatric hospitals: Accra Psychiatric, Pantang Hospital and Ankaful. Beyond the ring-fenced mental health budget, mental health spending is difficult to track in Ghana. This is in part due to the integration of mental health into primary care where the delineations of services offered are more difficult to capture. A key informant from a hospital in Ghana, states:

"The general perception of mental illness affects effective funding allocation. Is it a lost cause? Or can they be treated and be beneficial to society? People do not think about the loss of man-hours for the patients and their families who care for them."

Allocations to hospitals for the purchase of medicines vary year on year. Hospitals will sometimes supplement medicine shortages from out of their operating costs. Annual reports of the Pantang Hospital, for example, indicate that in 2007-2008 they received GH¢115,643 ($121,246) worth of psychiatric medicines, which represents an 82% decrease on the value of medicines received the previous year. As a result, in 2008 Pantang had to supplement its stock of psychiatric medicines by paying GH¢ 21,102 ($22,124), which is double the amount they contributed in 2007.

### Uganda

#### Public Funding for Mental Health

Uganda's national ring-fenced budget for mental health care in 2007-2008 was $14,178,880, which breaks down to a per capita allocation of $0.56 and represents 6.6% of Uganda's national health budget. It is important to note that all mental health allocation estimates in Uganda are based on a Ministry of Health key informant interview. According to key informants, mental health in Uganda is predominantly funded at the provincial level, rather than nationally. Uganda recently completed a WHO AIMS study, which found that 1% of the country's health budget was allocated to mental health in primary care. The 1% does not figure in the national ring-fenced budget for mental health, so it most likely comes from the provincial budgets.

#### Other Funding for Mental Health

**Figure 3:**
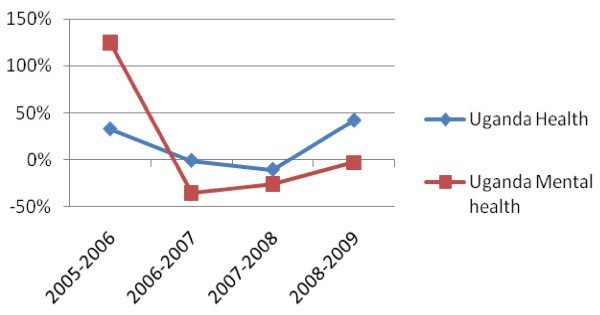
**Uganda % Change in Ring-Fenced Budget for Mental Health vs All Health 2005-2009**. Note: The Uganda data only accounts for the budget of Butabika Hospital and the National Department for Mental Health. It does not include the budget for community mental health, because trend data was not available.

In 2005, a large one-off donation was made by the African Development Bank in support of mental health. That year, the African Development Bank spent 45% of its health budget to Uganda on mental health. Over the past three years, Uganda's national mental health budget has been steadily increasing in parallel with its overall budget for health (refer figure 3).

Health insurance is another influential factor on mental health financing in Uganda. Private health insurance providers, such as African Air Rescue and International Air Ambulance, provide some coverage from out-of-pocket expenses for middle and upper class citizens. However, these insurance providers do not cover severe psychiatric and neurological disorders.

#### Budget Allocation

The large majority (85%) of Uganda's national ring-fenced mental health budget is allocated to its single public mental hospital, Butabika. The remaining $2.5 million (15%) of ring-fenced funds go to other mental health services, including community mental health care. As stated earlier, an additional 1% of Uganda's health budget is allocated to mental health in primary care, as reported by the WHO AIMS study.

Some key informants from Uganda felt that allocated mental health budgets were not disbursed properly. In one instance, the allocated budget was reportedly not spent. The informant from the Buliisa sub-district noted that in 2008/2009 351,684,208 Ugandan shillings (US $207,494) were allocated to mental and reproductive health and only 138,000 shillings (US $81) - less than 0.1% - were actually used. The informant states, "*I have never held a home visit or community outreach clinic for mental health for all the three years that I have been here yet fund allocations are always indicated in the budget. The funds never get disbursed." *In another instance, budgets were used but in ways deemed locally inefficient, because costly interventions were privileged over cost-effective ones. One key informant commented, "*An ambulance and accompanying staff are financially facilitated to transport a patient from upcountry to [the hospital] to get a largactil [antipsychotic] injection when it would be much cheaper to make the drug more accessible."*

### Kerala, India

#### Public Funding for Mental Health

**Figure 4:**
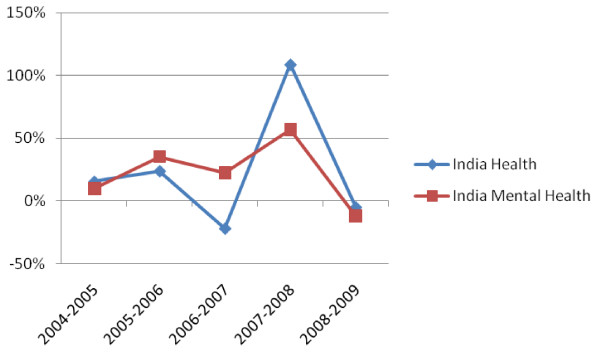
**India % Change in Ring-Fenced Budget for Mental Health vs All Health 2005-2009**.

In Kerala, $556,416 was ring-fenced to mental health care, which breaks down to a per capita allocation of $0.02, representing 2% of the State health budget. In India as a whole, the ring-fenced mental health budget has increased and decreased within the last 5 years consistently, although not exactly proportionately, with the health budget (refer figure 4).

#### Budget Allocation

The large majority (78%, US $432,200) of Kerala's ring-fenced budget went to three public mental hospitals: Thrivananthapuram, Kozhikode and Trichur. The remainder (22%, US $124, 200) was allocated to community-based care and mental health promotion. Efforts are in progress to develop a software that enables the State and the public to monitor more closely how mental health funds are spent in Kerala.

The community care component of Kerala's mental health budget constitutes part of India's District Mental Health Programme, launched in 1995 with the purpose of integrating mental health into four levels of the health system: community health centres, primary care, district hospitals and psychiatric units in medical colleges. Kerala has been cited as an example of best practice within that programme, in particular the district of Thiruvananthapuram (WHO WONCA 2008). The District Mental Health Programme first began in Kerala in 1999 funded by a five-year grant from the national government, through the National Mental Health Programme. The programme is now funded by the State of Kerala.

One stipulation of the grant was that the State would adopt responsibility for the funding upon termination of the five year grant. The State initially delayed funding. Recently, however, the State of Kerala allocated 2.5 million rupees (US $61,600) to the District Mental Health Programme for the fiscal year 2008/09. This achievement is thanks in part to lobbying by the Alliance for Mental Health Promotion, which was established by that very programme. This additional funding is not yet secured for the long-term, however, and some perceive that the money is not being spent. According to a key informant from the State Planning Board: "*There is no demand from the Districts for funds for mental health services; rather, the allotted funds are not utilized fully."*

### Sri Lanka

#### Public Funding for Mental Health

All quantitative data for Sri Lanka is taken from estimates relayed by government key informants. The researcher was not able to review actual government budgets to confirm these numbers. According to key informants in the health ministry, the total national ring-fenced budget for mental health in Sri Lanka in 2008/09 was $8,473,392. This amounts to US $0.44 per capita and represents 1.7% of the national health budget.

#### Other Funding for Mental Health

According to key informants, the World Bank matched the government funding of mental health. Together, the government and World Bank accounted for 90% of national mental health funding in 2008/09. The United Nations Population Fund and WHO comprised the remaining 10% of the mental health budget (see Table 1).

**Table 1:** Mental Health Funding for Prevention and Promotion in Sri Lanka 2008/2009

Funding Source	LKR	US $	% Budget
Government Treasury	100,000,000	909,000	44.8%
World Bank	100,000,000	909,000	44.8%
UN Population Fund	15,000,000	136,350	6.7%
World Health Organization	18,000,000	72,720	3.4%

Total	1,917,000	2,027,020	100%

1 LKR = 0.00909 USD

In Sri Lanka, health is budgeted at both national and provincial levels. According to estimates provided by the Ministry of Healthcare and Nutrition, provincial health budgets comprise 28% of the total health budget for Sri Lanka. The current study was not able to track mental health budgets at the provincial level.

#### Budget Allocation

The large majority (76%) of the national ring-fenced mental health budget in Sri Lanka is spent on 3 psychiatric hospitals: Angoda, Mulleriyawa and Handala. The remainder (24%, US $2 million) is allocated for mental health outside of psychiatric hospitals, namely to a national programme of prevention and promotion. Just over half of the budget for prevention and promotion comes from sources external to the government (see Table 1), whereas the psychiatric hospital budget is entirely government funded.

General hospitals also allocate funds to mental health on a case by case basis, which the researcher was not able to track through this study. Both national and provincial funding for mental health feeds into these institutional budgets. There currently appears to be no system in place to evaluate budget allocations and spending in relation to those allocations.

### Lao PDR

#### Public Funding for Mental Health

Lao PDR has no mental hospital and no ring-fenced budget for mental health. People with mental illness are seen in one of two general hospitals in Lao: Mahosot and Hospital 103, which is a military hospital. Budgets for the mental health units of these two hospitals are integrated within the overall hospital budgets, so the government expenditure on mental health could not be calculated. Government key informants did not disclose any further budget information to the researcher in Lao PDR and no information on mental health is available publicly. The WHO ATLAS project was also unable to secure financial data on mental health for Lao PDR. The most comprehensive published information about mental health in Lao PDR is a situation analysis conducted in 2002, which does not include financing [16].

#### Other Funding for Mental Health

BasicNeeds operates the only ongoing non-governmental mental health programme in Lao, based in the capital Vientiane and working with 1,583 people with mental illness or epilepsy and an estimated 800 carers. In the fiscal year 2007/08, BasicNeeds spent $197,783 in Lao PDR to implement the Model for Mental Health and Development. Some additional funding for mental health came from the WHO and the Belgian arm of Handicap International. The Handicap International Belgium funding was for a one-year pilot project. Funding supported the health clinic at the district hospital and, more specifically, self-support for victims of unexploded ordnance (UXO). No other international NGOs are known by BasicNeeds to be working in mental health in Lao PDR (Refer to Table 2).

**Table 2:** External Funding for Mental Health in Lao PDR 2007/08

Funding Source	US $
BasicNeeds	197,783
WHO	6,370
Handicap International	1,976

Total	206,129

### Cross-Country Comparison

Table 3 compares study findings on ring-fenced budgets for mental health care in each region.

**Table 3:** Mental Health Financing Data Cross Area Results (USD)*

(2007-08)	Ghana	Uganda	Kerala	Sri Lanka**	Lao
Total Population (WHO)	23,008,000	29,899,000	31,900,000	19,207,000	5,759,000
Government health budget	$450,252,725	$252,673,400	$27,820,800	$490,665,852	NA
Government expenditure as % of total health expenditure (WHO)	34%	29%	19%	46%	20%
Ring-fenced budget for public mental hospitals	$17,412,263	$14,160,000	$432,216	$6,466,392	NA
Number of public mental hospitals	3	1	3	3	0
Other ring-fenced budgets for mental health	None	$2,545,614	$124,200	$2,027,000	None
Total ring-fenced budget for mental health	$17,412,263	$16,705,614	$556,416	$8,473,392	0
Per capita ring-fenced budget for mental health	$0.76	$0.56	$0.02	$0.44	None
Ring-fenced budget for mental health as % of health budget	3.9%	6.6%	2.0%	1.7%	NA
Hospital budget as percentage of ring-fenced budget for mental health	100%	85%	78%	76%	NA
Government mental health spending per capita	$0.76	$0.56	$0.02	$0.44	NA
BasicNeeds Budget	$651,660	$329,643	NA	$478,126	$197,783

*Conversions are taken from http://www.oanda.com/currency/historical-rates

(USD 1 = GHS 1.04845, INR 0.02484, LAK 0.00011, LKR 0.00909, UGX 0.00059)

**Figure 5:**
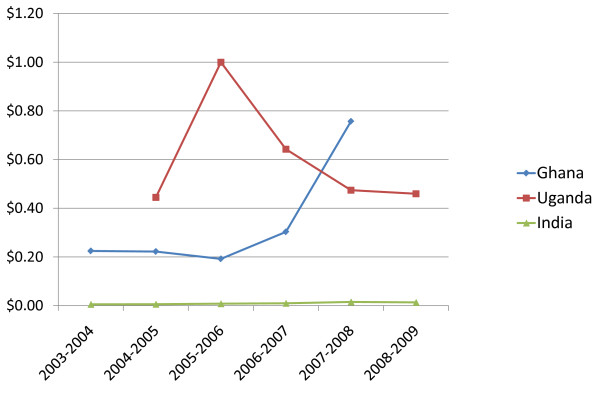
**Per Capita Government Ring-Fenced Spending Trends on Mental Health (USD)**.

The study also captured trends in government mental health budgets for Uganda, Ghana and India as shown in Figure 5. The figures represent national level spending, including for India; State or provincial level spending is not included. Table 4, Table 5 & Table 6 show the absolute figures behind the graph.

**Table 4:** Trends in the Public Mental Health Budget in Ghana

	GHS	Xchg Rate	USD	Per Capita	Increase
**2003-2004**	4,304,704	1.2	$5,165,645	$0.22	
**2004-2005**	4,645,511	1.1	$5,110,062	$0.22	-1%
**2005-2006**	4,017,096	1.1	$4,418,806	$0.19	-14%
**2006-2007**	6,338,300	1.1	$6,972,130	$0.30	58%
**2007-2008**	16,607,624	1.04845	$17,412,263	$0.76	150%

**Table 5:** Trends in the Public Mental Health Budget in Uganda*

	UGX	Xchg Rate	USD	Per Capita	Increase
**2004-2005**	23,744,000,000	0.00056	$13,296,640	$0.44	
**2005-2006**	53,375,000,000	0.00056	$29,890,000	$1.00	125%
**2006-2007**	34,306,000,000	0.00056	$19,211,360	$0.64	-36%
**2007-2008**	24,032,000,000	0.00059	$14,178,880	$0.47	-26%
**2008-2009**	24,100,000,000	0.00057	$13,737,000	$0.46	-3%

* This data excludes the budget for community mental health, which in 2008 represented 15% of the budget for mental health.

**Table 6:** Trends in the Public Mental Health Budget in India*

	INR	Xchg Rate	USD	Per Capita	Increase
**2003-2004**	280,000,000	0.02181	$6,106,800	$0.01	-
**2004-2005**	300,000,000	0.02233	$6,699,000	$0.01	10%
**2005-2006**	400,000,000	0.02266	$9,064,000	$0.01	35%
**2006-2007**	500,000,000	0.02216	$11,080,000	$0.01	22%
**2007-2008**	700,000,000	0.02484	$17,388,000	$0.02	57%
**2008-2009**	700,000,000	0.02182	$15,274,000	$0.01	-12%

* This is the budget of the National Mental Health Programme, not Kerala.

Ghana currently has the highest per capita spending on mental health of the three countries providing data ($0.76), but over the past five years as a whole, Uganda has had a higher level of spending. The mental health budget in Uganda has vacillated, largely as a result of the African Development Bank donation in 2005, whereas in Ghana and India it has been steadily on the rise in tandem with the overall health budgets. Part of the explanation for the low rate of public spending on mental health in India ($0.02) as compared to Uganda and Ghana is that public funds represents a considerably smaller portion of overall health spending in India (19%) than in the two African countries (29% and 34% respectively). Four out of five health care dollars in India are paid out-of-pocket [12]. Another factor could be the underutilization in India of the National and District Mental Health Programme funds, including states not applying for these funds.

## Discussion

### Insufficient and Inconsistent Funding for Mental Health Coverage

#### Public Sector

Public funding for mental health fell short of need in all five countries studied. In the four countries with a mental health budget (all but Lao PDR) allocation to mental health ranged from 1.7% to 6.6% of the national health budget. The annual government expenditure per capita ranged from US $0.02 to US $0.76 per capita. This is 2 to 100 times less than the amount necessary ($1.85 per capita) to achieve desired levels of mental health coverage [8]. The $1.85 per capita recommended by Chisholm and colleagues is an aggregate figure, whereas needs and costs differ on a country by country basis, however it nonetheless gives an idea of the order of magnitude of spending necessary in low-to-middle income countries.

Although public mental health budgets are too low to meet needs in the countries studied, there is nonetheless an encouraging trend towards increased spending in the three countries who made such data available (India, Ghana and Uganda). While in India and Uganda, mental health budgets have risen with the tide of overall health budgets, Ghana is an exception, where an increase in the mental health budget between 2007 and 2008 coincided with a decrease in the overall health budget. These encouraging trends should be built upon over the next five years in order to start achieving the desired and necessary levels of service coverage.

#### Multilaterals and Aid Agencies

Outside of the national treasury, multilaterals and aid agencies also contributed to mental health budgets in the countries studied. The most significant multilateral contribution observed in this study was made by the African Development Bank, which in 2005 allocated 45% of its health budget for Uganda to mental health. This was part of a one-off grant donation supporting reproductive health, maternal and child care services and mental health services. Another significant donor to mental health has been the World Bank, particularly in Sri Lanka, where it paid for 10% of the national government's ring-fenced mental health budget, all of which went to promotion and prevention. Smaller donors are the WHO and the UN Population Fund (FPA), both of which contributed approximately 1% of the national mental health budget in Sri Lanka in 2008. While these contributions made an impact, they are not always consistent and cannot replace national budgeting for mental health. The examples of the African Development Bank and the World Bank need further exploration to determine how the money was raised and spent, and importantly how such funds can contribute more meaningfully to longer term impact on financing practice.

#### Private Funding

According to WHO estimates, private spending accounts for the majority of health dollars in all five of the countries studied, with government funds averaging only one third (32%) of the total health expenditure [17]. Indeed, government clinics are not always free of charge. Thus, while governments represent the majority of mental health service provision (outside of traditional healers) they do not represent the majority of mental health service funding.

Little is known about private funding of mental health services in developing countries. Broadly speaking, private services can be divided into two categories: the for-profit and the not-for-profit, the latter of which are predominantly non-governmental organisations (NGOs). In the areas studied, BasicNeeds was the largest NGO working in mental health, though not the only NGO. The authors had access to financial data from BasicNeeds, but not from other NGOs. Despite the incompleteness of this data, the budget of BasicNeeds in the countries studied can serve as a lens into the order of magnitude of NGO spending on mental health.

The average per annum expenditure of BasicNeeds in Ghana, Uganda, Sri Lanka and Lao PDR was $414,000. This represents an average of 3.8% of the government budgets for mental health in the four countries with mental health budgets. The NGO and government budgets are not comparable in terms of activities, however the numerical comparison lends an idea of the level of funding from international private agencies on mental health. Since the focus of BasicNeeds is on certain districts rather than on the country as a whole, it has a greater impact at the district level than nationally. The NGO leverages its investment by rallying the support of government - both health and social welfare - for mental health service provision. Indeed, funding for the NGO has tended not to come from health agencies, but rather via funding streams in support of poverty reduction and disability rights.

#### Cost-Effectiveness and Data

One potential reason for the underfunding of mental health care is the perception that it is not cost-effective. A key informant from Ghana expressed this view, asserting that some health professionals view mental illness as a "lost cause." Insufficient funds, in turn, undermine the effectiveness of mental health interventions, creating a negative feedback loop.

Cost-effectiveness should be a key criterion for decisions about budget allocation, according to step 4 of the WHO Mental Health Financing guidelines. Indeed, the WHO has spear-headed an initiative to increase data on the cost-effectiveness of health interventions, including mental health interventions, in a project called CHOICE (Choosing Interventions that are Cost-Effective) [18]. More local data is needed on the cost and effectiveness of mental health interventions, so as to make a sound economic case for mental health care. However one of the reasons for the lack of appropriate data is that field programmes implementing community mental health, from where data can be drawn, are not that many.

It is important too that calculations of cost effectiveness cover not only treatment services provided in a clinic or primary care, but include also cost of items which are important to effective and sustainable community based mental health practice. Therefore cost effectiveness studies should consider costs related to appropriate human resource development e.g. costs related to training health personnel at different levels, taking into account too some recurring challenges in LMICs such as frequent transfer of trained health staff, poor follow up in the community. So other costs such as training of and salaries for community based workers (whose services are crucial to sustaining effective community interventions), making home visits and other follow up in the community become important. In short current understanding about cost effectiveness has to broaden so as to include those costs which are fundamental to effective community based mental health.

#### Unspent money

Insufficient funding for mental health is not only the result of budgeting. It can also stem from problems in distributing or spending budget allocations. Such was the example brought to our research officers in Buliisa, Uganda, where less that 1/10^th ^of a percent of the budget allocated to mental and reproductive health in 2008 was spent, according to the records. One possibility in this case is that the funding was spent but not accurately recorded, since research officers noted from qualitative evidence that some public funds had been spent on mental health. Unspent budgets were also a concern raised by key informants in Kerala. Given the general shortage of funds for mental health, the reasons for these distribution problems need to be explored.

### Disconnect Between Mental Health Policy and Financing Practice

#### Policies promoting community approaches

Over the last decade, mental health policy in low and middle-income countries has increasingly focused on community-based, rather than hospital-based care. At the international level, the World Health Organization has been a vocal proponent of community care in reports such as *The World Health Report 2001 *[19], *the Mental Health Gap Action Programme *[20] and *Integrating Mental Health into Primary Care *[21]. Nationally, the rhetoric has also shifted in most of the countries studied towards community-based care. This is witnessed in India's District Mental Health Programme (1995), Ghana's new mental health bill, which stands for ratification by parliament, and the draft mental health policies of Uganda and Kenya [22]. Given the near consensus of mental health policies for providing community-based alternatives to institutional care, the actual allocation of public mental health funds reveals a striking disconnect between policy and practice.

#### Allocation to hospitals

In all four countries that tracked mental health spending, the vast majority of the national mental health budget allocation (a range of 76% to 100%) went to psychiatric hospitals. In Ghana, psychiatric hospitals were the only budgeted mental health service. Sri Lanka and Kerala both have three psychiatric hospitals to which they each devote three quarters of their national mental health budgets. Uganda has only one psychiatric hospital (Butabika), to which it devotes 85% of its national budget for mental health. Lao has no psychiatric hospital and no ring-fenced budget for mental health.

Over-reliance on psychiatric hospitals, leads to custodial rather than rehabilitative care, and an insufficient choice in treatment [23]. Moreover, increased expenditure on psychiatric hospitals does not necessarily lead to wider access to services, since most psychiatric hospitals are located in urban centres and inaccessible to rural populations, who represent the majority in the five countries studied.

#### Allocation to community-based care

Despite the preponderance of funding for institutional care, three countries nonetheless invested some ear-marked public funds in community mental health. Integrating mental health into primary care has been the main emphasis of advocacy for community care. The WHO AIMS study in Uganda states that 1% of the health budget ($2.5 million) has been ring-fenced for mental health in primary care [24]. Community mental health also means health promotion, out-reach and building social capital by working with lay-members of the community. Sri Lanka is spending 0.4% of its national health budget ($2 million) on prevention of mental illness and promotion of mental health. Thanks to India's promising District Mental Health Programme, which has been cited as a case of best practice in integrating mental health into primary care [20], 0.4% of Kerala's health budget ($120,000) has been allocated to community out-reach for people with mental health problems. Non-government agencies, such as BasicNeeds, have also been particularly strong advocates of community-based care, and are supported in this endeavour by the grass-roots lobbying of self-help groups. These initiatives are a promising step in aligning policy with budget allocation, but as of yet they remain the exception rather than the rule.

#### Allocation to medicines

Insufficient funding for mental health contributes to crippling shortages in psychiatric medicines. Budgeting processes for psychiatric medications differ from country to country, but in all the countries studied, key informants complained of persistent shortages in the public supply of essential psychiatric medicines, leading alternately to heavy out-of-pocket costs or discontinued treatment.

In Ghana, the Chief Pharmacist of a given psychiatric hospital collates consumption patterns and creates an annual budget for procurement, which is reviewed by the Director for Procurement and Supplies at the MOH and approved by the Ministry of Finance and Economic Planning. This lengthy budgeting process does not allow for flexibility in response to shortages in drug supply. Moreover, the drug budget is subject to variation year on year and requires supplementing by hospitals and private funders, as demonstrated in the case of Pantang Hospital, which contributed $22,124 in 2008 to supplement medicine shortages.

In Uganda, two budget lines fund the supply of psychiatric medicines: the primary health care budget and the Credit Line budget. Whereas the primary care budget is managed at district level, the Credit Line system is managed at the health centre. In both instances, negotiations take place with the National Medical Stores, who distribute the drugs. In Buliisa district, key informants estimated that 50% of the funding going to mental health was being spent on drugs, however it was noted that precise numbers are difficult to estimate. No data was available on the budget for psychiatric medicines in Kerala, however in Gujarat, 20% of the District Mental Health Budget is allocated to psychiatric medicines and supplies.

Out-of-pocket payments are the solution of last resort when public medicine supplies run short. In Uganda, key informants noted that drugs were being sold in health centres, which defies the national health policy of free medicines. Moreover, drugs sold to individuals are often priced at a higher rate than to clinics. In Lao PDR, where there is no budget for mental health, people pay out of pocket for psychiatric drugs. The cost of antipsychotic medicines was estimated at US $17 (145,000 kip) per month. Informants speculated that the high cost causes a substantial number of people to discontinue treatment. Sudden withdrawal or substitution of medications in times of shortage can have very negative effects on a person's health. It is therefore essential that systems be put in place to flexibly measure and meet demand for essential psychiatric medicines.

#### Horizontal allocations

The recent trend in health budgeting has been towards horizontal spending, in other words spending to strengthen the whole health system, rather than investing vertically in a specific disease. In this context, mental health is often grouped with other health budgets, such as the rural health budget in India (the National Rural Health Mission) and the reproductive health budget in Uganda. Sri Lanka has two budget groups for mental health: curative health care and promotional and preventive health care.

Integration of mental health into other budgets has both gains and pitfalls. From a financial perspective, when integrated into primary care, mental health resources are not always clearly tracked and there is a danger that funds go unallocated to interventions that benefit mental health. However, at times integrated budgets benefit mental health allocations, particularly at the hospital level. One key informant from Ghana noted that in district hospitals it was to the advantage of people with mental health problems that the hospital pooled its funds across conditions. This is because people with mental health problems tend to be more poor and unable to pay the fees, called internally generated funds, which support the hospital's operation. Thus, people with other health conditions subsidise the mental health unit. More broadly, spending on the overall health system may benefit mental health along with other health conditions.

## Conclusions

### Limitations

The study results include many limitations. One major limitation is that researchers were only able to collect data on ring-fenced budgets for mental health rather than tracking all spending related to mental health. This data was only available at national level, with the exception of Kerala, where it was available at State level; but much health expenditure is budgeted at provincial level, particularly primary care. Ring-fenced spending for mental health also excludes more difficult to track resources outside of the health sector, such as within social welfare and education, which can have a great impact on mental health. Finally, ring-fencing misses out spending by individuals and households, who, in the absence of decent public services, take it upon themselves to purchase a mix of informal and formal health care in the private sector, including traditional healing. There are therefore significant other sources of funding for mental health that are not represented in this data.

Our private sector data is similarly limited by what we had access to given our research capacity. Thus, for-profit private sector mental health services were excluded from analysis, and non-profit budgets were presented primarily for BasicNeeds.

Cross-country analysis is also problematic for a number of reasons. The selection of countries was a convenience sample based on where BasicNeeds researchers have a presence and knowledge of key informants. The only logic for comparison across these geographies is that they are low and middle income countries grappling with similar challenges of scarce resources for mental health. Further anthropological or sociological research could shed light onto the cultural contexts, political economy and specific financing challenges in each of these places. In addition, the use of a top-down approach to mental health funding as pursued here will always be problematic at an international level of comparison as long as there is no standard reporting requirement for countries to heed, as they now do for HIV/AIDS, malaria and other high-profile diseases.

Finally, data collection was limited by the lack of recorded data on mental health financing, and an occasional lack of transparency. All quantitative data from Sri Lanka, for example, is based upon key informant estimates as the actual budgets were not provided to the researcher. However, since the study aims in part to illuminate barriers to tracking mental health spending in each country, many of these limitations are themselves informative.

### Recommendations

The following recommendations are not prescriptive but are meant to be viewed as pointers leading to more concrete steps:

1. A system of national health sub-accounts should be established for a defined set of mental health services. Funds in a sub-account could be used only for the purpose of the mental health service defined, for example mental health in primary care. This would enable better tracking of expenses and ensure that mental health is adequately budgeted.

2. Government budgets for mental health should be based on comprehensive assessments of service needs and intervention costs specific to their location. The current paucity of such data, however, cannot continue to be a reason for inaction or indeed inappropriate action. Initial estimates of needs and cost may be estimated based upon a common framework for community based interventions in LMICs, developed from currently available evidence.

3. Public spending on mental health should be tracked by national health information systems and made accessible to the public. The Kerala state initiative to track mental health spending and make this information open to the public is commendable and it is recommended that other governments follow this example. Budget tracking should include information on allocation, actual dispersal, utilization and, where possible, be linked to performance.

4. Funding for mental health should be de-centralised and become part of district annual budgets. Local government bodies in collaboration with locally formed user groups or user representatives should monitor the utilization of such public mental health funds.

5. Funds for mental health interventions from multilateral institutions and other aid agencies should be distributed over the mid-to-long term to build the mental health system, rather than in one-off donations for short-term projects.

6. Governments should adopt the WHO AIMS indicators for reviewing national mental health finances. This will provide a better idea of the magnitude and nature of funding gaps and thus a stronger basis for planning budgets. Adopting WHO AIMS financing indicators will also enable cross-country comparison, as is possible with HIV/AIDS and malaria.

7. Partnerships between the public and private sectors can significantly increase the funding for mental health and the reach of community-based interventions. Data generated from such interventions can further refine intervention models and inform future budgetary considerations.

Informed decisions about how to build mental health resources are made possible by accurate mental health intervention, costing and utilization data. In the countries studied, this data, along with systematic recording processes from the national level down to the grass roots level are not currently available to internal decision-makers, as well as to the public. So it is important that these become available. Even more important, mental health financing should be infused with fresh thinking that encompasses new ideas emerging from community based interventions. This can enable effective scale up aimed to bring qualitative improvement to the lives of people with mental illness or epilepsy in LMICs.

## Competing interests

The authors all work for BasicNeeds, a non-governmental organisation promoting mental health and development in the five countries studied.

## Authors' contributions

All authors have read and approved the final manuscript. SR provided comprehensive feedback and final approval on the research study design, analysis and article development. SKW contributed to the research study design, analysis and article development. She provided drafts of the results and conclusions sections. VdM contributed to the analysis and article development. She provided drafts of the background, results and discussion sections. SCM carried out data collection and analysis for research completed in India. He also drafted the methodology section of the article and provided literature review and referencing support.
